# Bridging Neuroimaging and Neuropathology: A Comprehensive Workflow for Targeted Sampling of White Matter Lesions

**DOI:** 10.1111/jon.70094

**Published:** 2025-10-22

**Authors:** Nadim Farhat, Jinghang Li, Jacob Berardinelli, Mark Stauffer, Andrea Sajewski, Salem Alkhateeb, Noah Schweitzer, Hecheng Jin, Sossena Wood, Milos D. Ikonomovic, Jr‐Jiun Liou, Howard J. Aizenstein, Joseph M. Mettenburg, Tales Santini, Minjie Wu, Julia K. Kofler, Tamer S. Ibrahim

**Affiliations:** ^1^ Department of Bioengineering University of Pittsburgh Pittsburgh Pennsylvania USA; ^2^ Center for Data & Computational Science, Veterans Affairs Boston Massachusetts USA; ^3^ Department of Pathology University of Pittsburgh School of Medicine Pittsburgh Pennsylvania USA; ^4^ Department of Biomedical Engineering Carnegie Mellon University Pittsburgh Pennsylvania USA; ^5^ Department of Neurology University of Pittsburgh School of Medicine Pittsburgh Pennsylvania USA; ^6^ Department of Psychiatry University of Pittsburgh School of Medicine Pittsburgh Pennsylvania USA; ^7^ Department of Radiology University of Pittsburgh School of Medicine Pittsburgh Pennsylvania USA

**Keywords:** MRI‐guided histology, neuroimaging workflow, postmortem brain imaging, three‐dimensional (3D) printing, ultrahigh field MRI, white matter lesions

## Abstract

**Background and Purpose:**

White matter lesions are common imaging biomarkers associated with aging and neurodegenerative diseases, yet their underlying pathology remains unclear due to limitations in imaging‐based characterization. We aim to develop and validate a comprehensive workflow enabling precise MRI‐guided histological sampling of white matter lesions to bridge neuroimaging and neuropathology.

**Methods:**

We established a workflow integrating agar‐sucrose brain embedding, ultrahigh field 7 Tesla (7T) MRI acquisition, reusable three‐dimensional (3D) printed cutting guides, and semiautomated MRI‐blockface alignment. Left hemispheric postmortem brains were stabilized in the embedding medium and scanned using optimized MRI protocols. Coronal sectioning was guided by standardized 3D‐printed cutting guides, and knife traces were digitally matched to MRI planes. White matter lesions were segmented on MRI and aligned for histopathological sampling.

**Results:**

The workflow enabled reproducible brain sectioning, minimized imaging artifacts, and achieved precise spatial alignment between MRI and histology. For demonstration, detailed results from two representative brains were presented in this article. Consistent, high‐resolution MRI data facilitated accurate lesion detection and sampling. The use of standardized cutting guides and alignment protocols reduced variability and improved efficiency.

**Conclusions:**

Our cost‐effective, scalable workflow reliably linked neuroimaging findings with histological analysis, enhancing the understanding of white matter lesion pathology. This framework held significant potential for advancing translational research in aging and neurodegenerative diseases.

## 1 Introduction

White matter lesions are among the most common aging and neurodegenerative disease biomarkers on brain MRI scans. These lesions appear as hyperintense regions on T2‐weighted MRI and hypointense on T1‐weighted (T1w) MRI. Broadly, these lesions can be explained by demyelination, axonal loss, and gliosis [1, 2, 3, 4, 5]. Clinically, white matter lesions are associated with aging, vascular risk factors (e.g., hypertension and diabetes), and neurodegenerative disorders [3, 6, 7, 8, 9, 10, 11]. An increased lesion burden is strongly linked to adverse clinical outcomes, including stroke, cognitive decline, dementia, and mortality [12, 13]. Despite their clinical significance, they remain poorly characterized at the histological level, limiting their utility for disease understanding and treatment development.

Current studies rely on ultrahigh field (UHF) MRI, typically defined as magnetic field strengths of 7 Tesla (T) and above, such as 7 and 9.4 T, for postmortem MRI‐guided histopathology to bridge the gap between imaging findings and microscopic pathologies [14, 15, 16, 17, 18, 19, 20, 21, 22, 23, 24, 25]. In our study, we utilized a human 7 T MRI system for image acquisition due to limited access to small‐bore preclinical scanners. Whole hemisphere human postmortem MRI at UHF human systems presents unique challenges. Conventional radiofrequency (RF) coils, optimized for in vivo imaging, produce inhomogeneous RF excitation for ex vivo specimens, often necessitating restricted fields of view or brain slab dissection to avoid signal dropout [26, 27]. Additionally, standardized protocols for brain embedding, sectioning, and MRI‐histology alignment remain underdeveloped. Although customized ex vivo imaging solutions exist, they typically require brain‐specific cutting guides that must be individually molded to each specimen's morphology [28, 29].

To address these challenges, we present a comprehensive, cost‐effective workflow integrating reusable three‐dimensional (3D)‐printed cutting guides, optimized agar embedding, and semiautomated MRI‐blockface alignment for precise histopathological sampling of white matter lesions. Our method ensures reproducible brain sectioning, minimizes MRI artifacts, and facilitates high‐accuracy registration between postmortem imaging and histology. By standardizing the process from MRI acquisition to tissue sampling, this workflow enhances the translational potential for postmortem studies, directly bridging imaging biomarkers and underlying neuropathology.

## 2 Methods

### 2.1 Workflow Description

**FIGURE 1:**
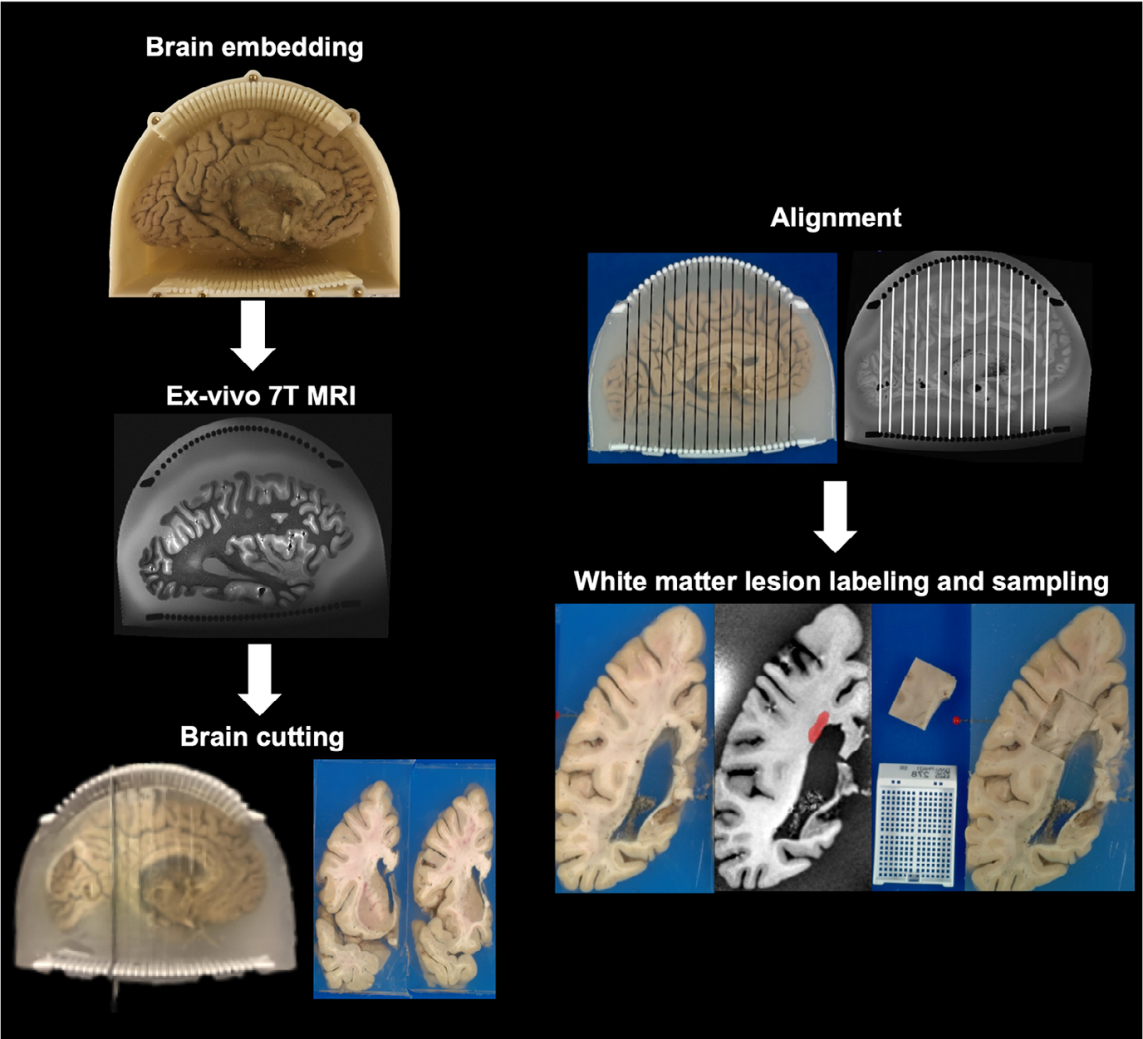
Workflow of MRI‐guided sampling of white matter lesion. The left hemisphere is first embedded in agar/sucrose solution and imaged using ultrahigh field 7 T MRI. The brain is then sectioned using a custom three‐dimensional‐printed cutting guide to ensure precise alignment with MRI slices. White matter lesions are identified on MR images, and corresponding tissue samples are extracted from the matched histological sections for further analysis.

Our methodology followed a systematic, multistep procedure designed to precisely identify and sample white matter lesions through UHF MR imaging, section cutting, and semiautomated alignment. Following our imaging bank's standardized protocol for postmortem brain processing, we utilized the left hemisphere from all postmortem human brains in this study. An overview of the entire workflow is provided in Figure 1, whereas detailed protocols for each step are described in the subsequent sections.

Brain preparation and embedding: We first prepared the postmortem brain and embedded it in agar gel. This stabilized the tissue during imaging and protected it from deformation during subsequent sectioning steps. The agar medium was carefully formulated with a sucrose mixture to optimize imaging quality.

UHF MRI acquisition: The embedded brain underwent scanning with UHF MRI at 7 T, which enables imaging of detailed brain structures at 370 µm isotropic resolution—a level of detail critical for precise alignment with blockface photographs and white matter lesion detection.

Guided brain sectioning: Following MRI acquisition, we sectioned the brain into coronal slabs using our custom 3D‐printed cutting guides. Each slab was approximately 9.8 mm thick, with the cutting guides ensuring consistent and accurate sectioning. Each slab surface was photographed to create blockface reference images.

Image alignment: We spatially aligned the coronal MR images to their corresponding blockface photographs using the distinctive patterns created by the cutting guides as reference points. These alignments were digitally preserved to maintain spatial correspondence between imaging and physical specimens.

White matter lesion sampling: The segmented white matter lesions, visible on both T1w and T2‐weighted sequences, guided precise histopathological sampling. Our in‐house deep learning model automatically identified these lesions on the T1w sequence, and the neuropathologist used these lesion masks with the saved MRI‐blockface alignments to target specific regions on the physical brain slabs for histopathological analysis.

### 2.2 Cost‐Effective Imaging Container

**FIGURE 2:**
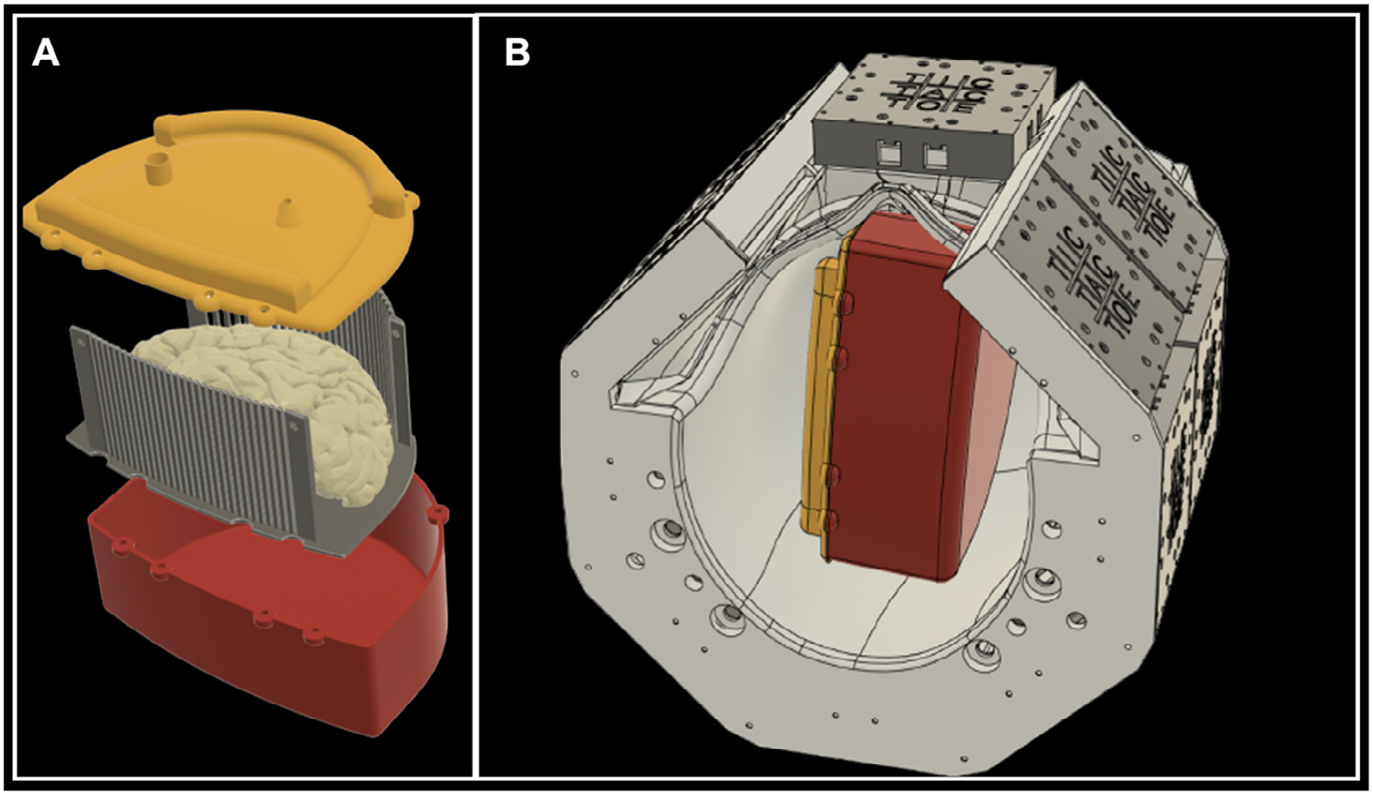
Three‐dimensional (3D) rendering of the 3D printed container with cutting guides. (A) The 3D model of the cutting guides (grey), the container enclosure (red), and the sealing lid (yellow). (B) 3D rendering shows the fit of the container to the RF coil's inner dimensions.

The imaging container consisted of an enclosure, a cutting guide, and a sealing lid. All three components could be manufactured via 3D printing. The enclosure protected the brain tissue and the embedding medium from deformation during scanning (red part in Figure 2A). The shape of the enclosure conformed to the inner shape of the receive coil (Figure 2B) and coarsely approximated the shape of a hemispheric forebrain. The entire enclosure's bounding box dimensions were as follows: 200 mm in the anterior–posterior direction; 100 mm in the medial–lateral direction; 165 mm in the superior–inferior direction. The enclosure featured 3 mm thick walls and base. The components of the enclosure were 3D printed using polycarbonate filament at 0.01‐in. layer resolution on a Fortus 450 3D printer (Stratasys, USA). This common engineering‐grade plastic provides high layer adhesion, impact resistance, and durability. Printing time for all components was approximately 42 h at approximately $60.00 in raw materials cost. The enclosure is specifically designed to fit within our in‐house developed MRI RF coil [30, 31, 32].

The cutting guides (grey part in Figure 2A) were designed to precisely control brain sectioning by restricting the knife's movement through the coronal plane. Conforming to the enclosure's shape, the guide consisted of parallel columns with precisely spaced gaps that direct the pathology knife. The structure included 28 top cylindrical columns arranged in an arc to match the brain's dorsal curvature, 29 bottom cylindrical columns in a gentle curve to accommodate the temporal cortex, 4 rectangular corner columns, and a base plate. The cylindrical columns (4 mm diameter) were positioned 0.6 mm apart, sized specifically to guide a standard 0.5 mm pathology sectioning knife. These columns spanned 129.4 mm from the prefrontal to occipital lobe, covering the typical extent of a left‐hemisphere brain.

The sealing lid (yellow part in Figure 2A) provided a secure seal for the enclosure while incorporating functional features for improved agar embedding. It featured an inlet port for adding additional agar after the enclosure was closed and an outlet port that enabled air suctioning during the filling process. This dual‐port design allowed for the removal of trapped air bubbles while simultaneously filling the container, improving the quality of the embedding medium.

### 2.3 Brain Embedding

To mitigate motion artifacts during scanning and protect the brain from deformation during handling and sectioning, we embedded postmortem human brains in an agar‐sucrose mixture. This medium not only provided structural support but also improved the signal‐to‐noise ratio and contrast‐to‐noise ratio compared to formalin‐fixed brains [33, 34].

For optimal UHF MR imaging, the embedding medium's dielectric properties must closely match those of the brain. Although the relative permittivity of water and agar is ∼78, the brain's permittivity is approximately 58 [35]. By adding sucrose to the agar solution, we reduce the medium's permittivity from 78 to 68, closely approximating the brain's dielectric properties at room temperature [15, 36].

Approximately 3000 mL hydrogel solution of 1.5% (w/v) agar (A5431, Millipore Sigma, Burlington, MA, USA) and 30% (w/v) sucrose (S5‐3, Fisher Chemical, Pittsburgh, PA, USA) was prepared [37, 38]. The agar‐sucrose mixture was then heated to 65–68°C on a hot stir plate over 2–3 h until fully dissolved, forming a clear solution. After turning off the heat, the solution was continuously stirred in a water bath until cooling to 42°C. It was then considered ready for brain embedding (Figure 1).

The brain was moved from 10% formalin to an empty container with the lateral side up, and warm agar‐sucrose solution was slowly pipetted to the gyri for full penetration of agar‐sucrose solution using a 10‐mL serological pipet (NC9868325, Fisher Scientific, Pittsburgh, PA, USA). A cutting guide was placed inside the embedding enclosure. The left hemisphere of the brain was positioned with the lateral side down and ventricles facing upward. The ventricle was first filled with agar, followed by careful pouring around the brain while massaging to eliminate air bubbles. The brain was gently pressed into a fully submerged position for 2–3 min until the agar thickened, preventing flotation. After 2–3 h, the agar fully solidified, forming a stable, transparent encasement. The enclosure was then sealed with the lid and was ready for imaging.

### 2.4 Postmortem Brain MRI Acquisition

**FIGURE 3:**
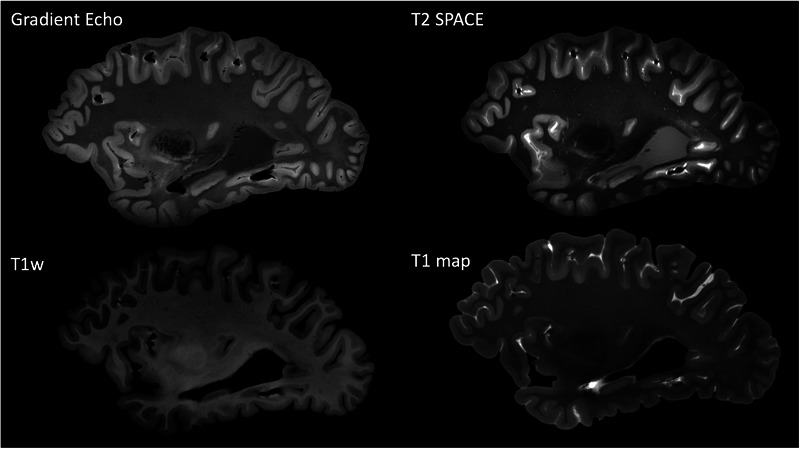
Example of acquired MR contrasts for postmortem brain MRI. T2 SPACE, T2 sampling perfection with application‐optimized contrast using different flip angle evolution; T1w, T1‐weighted image from magnetization‐prepared two rapid gradient echo (MP2RAGE) sequence; T1 map, quantitative T1 relaxation time map from MP2RAGE acquisition.

All postmortem MRI scans were acquired using a 7 T Siemens Magnetom scanner (Siemens Healthineers, Erlangen, Germany). During scan acquisition, the prepared container was placed inside our custom‐designed head coil, as shown in Figure 2B [30, 39, 40, 41, 42]. The brain was imaged using the following MRI pulse sequences: gradient echo (GRE): repetition time (TR) = 40 ms, echo time (TE) 1 = 8 ms, TE2 = 16 ms, 256 slices, voxel size = 0.37 × 0.37 × 0.37 mm^3^, acquisition time = 36 min and 59 s. T2 sampling perfection with application‐optimized contrast using different flip angle evolution (T2‐SPACE): TR = 3400 ms, TE = 368 ms, 208 slices, voxel size = 0.41 × 0.41 × 0.41 mm^3^, acquisition time = 46 min and 19 s. T1w magnetization prepared two rapid GRE (MP2RAGE): two GRE echoes, TR = 6000 ms, TE = 3.57 ms, TI1/TI2 = 556/2200 ms, flip angles = 6°/7°, 240 slices, voxel size = 0.37 × 0.37 × 0.37 mm^3^, acquisition time = 32 min and 28 s. Acquired image examples with the stated parameters are shown in Figure 3.

### 2.5 Brain Cutting

**FIGURE 4:**
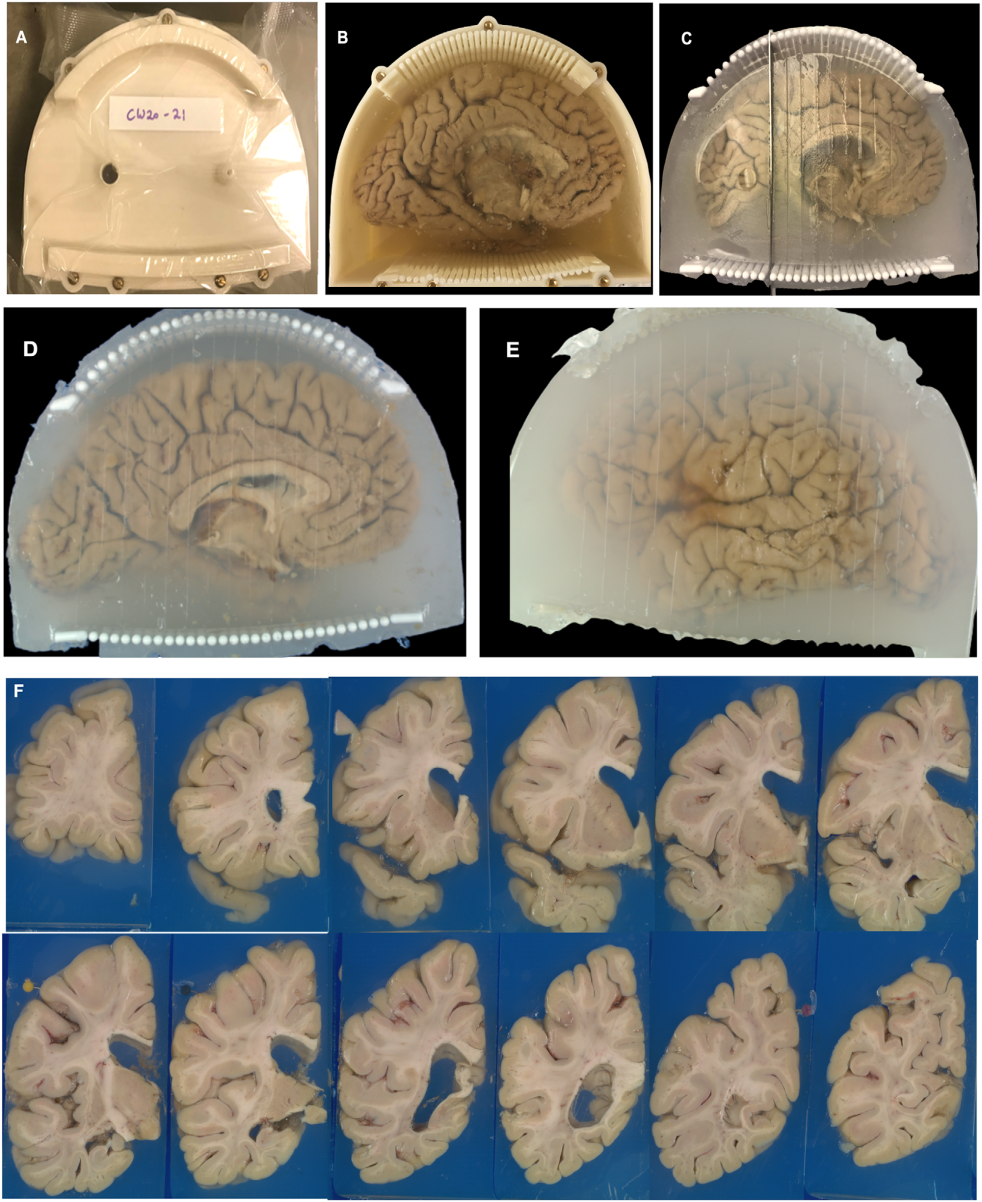
Brain cutting process. (A) The sealed container with the lid in place. (B) After removing the lid, the brain and cutting guide are visible, embedded in agarose. (C) The container is removed, and the brain is sectioned using a knife guided by the cutting columns. (D) The brain is fully sectioned; both the agarose and brain maintain their structural integrity. (E) The cutting guides are removed, leaving the slabs embedded in agarose. (F) The slabs are arranged and photographed.

Brain sectioning began by removing the lid of the enclosure (Figure 4A,B). Using the rectangular columns as handles (Figure 4B), the cutting guides were separated from the enclosure by first loosening the embedding medium along the enclosure walls with a knife. Once detached, the cutting guides along with the embedded brain were removed and placed flat on a working surface.

The neuropathologist started sectioning by aligning a 12‐in. brain knife (CellPath #CAA‐1201‐01A, 0.5 mm thick) between the top and bottom columns of the guide, corresponding to the planned initial coronal cut (Figure 4C). Subsequent slicing of the left hemisphere brain was performed by inserting the knife between cylindrical columns of the cutting guide—skipping two column pairs per cut—to maintain alignment with the original orientation (Figure 4D). After completing the sectioning, the knife marked on the surface of the embedding medium were photographed. These cut traces were critical for reconstructing the cutting planes and aligning postmortem brain MRI slices during multiplanar reconstruction (MPR) (Figure 4E).

In some cases, portions of the brain extended beyond the spatial coverage of the cutting guides. Due to the brain's fixation in the embedding medium, it was not possible to shift its position to align with the guiding columns. Consequently, these portions of the brain needed to be freehand sectioned without the guiding columns, while ensuring cuts remained parallel to the guided sections.

Finally, although the alignment of MR images and block face photographs (Figure 4F) for targeted tissue sampling was being processed and generated, the brain slices were temporarily removed from the container and placed back in 10% formalin for up to a week.

### 2.6 Ex Vivo MRI to Blockface Photos Alignment and White Matter Lesion Sampling

**FIGURE 5:**
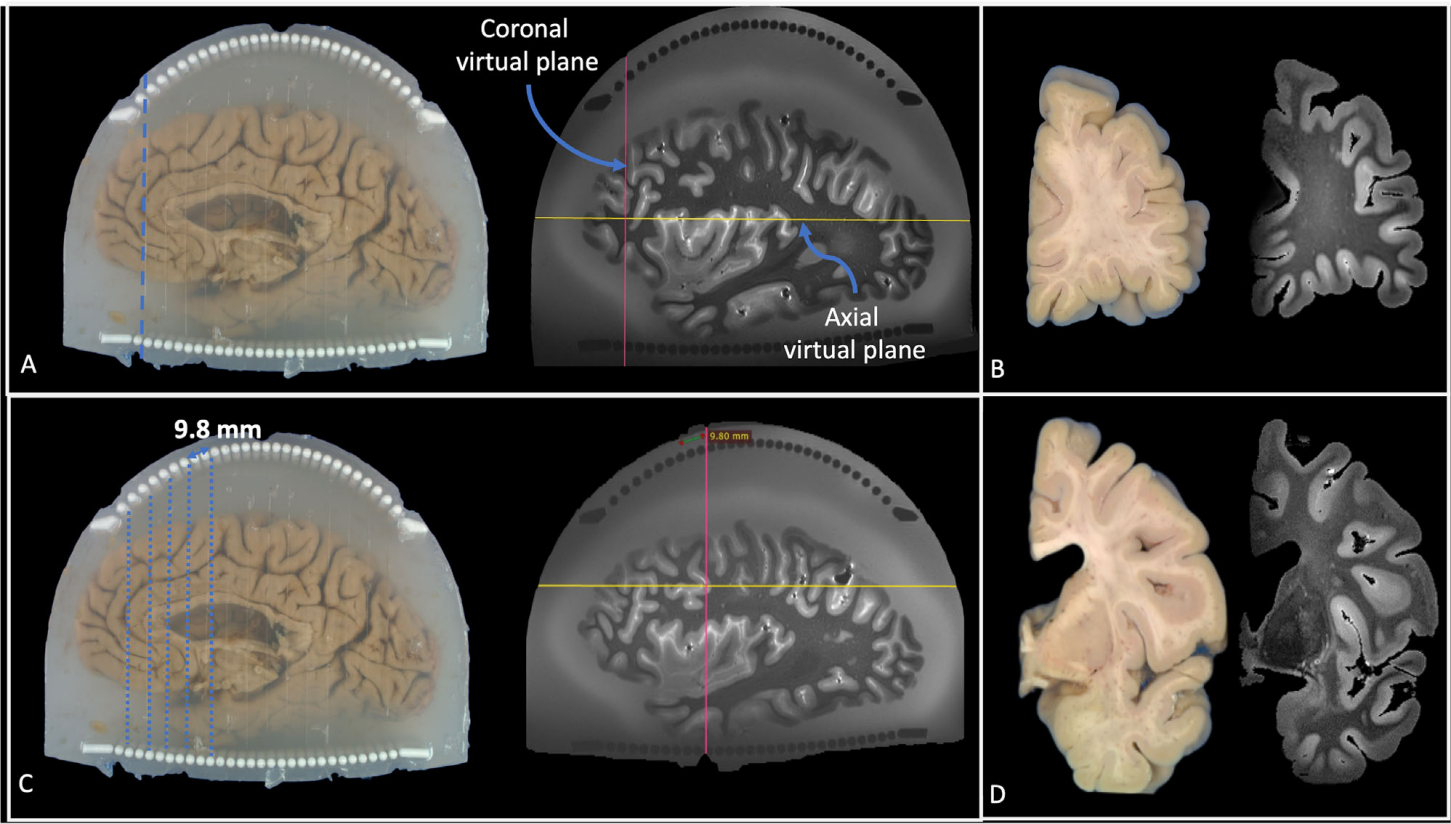
Alignment of MRI virtual cutting planes with knife traces on blockface photographs. (A) After sectioning the brain using the cutting guides, knife traces become visible on the agarose surface and are manually highlighted with a dashed blue line. The pink line represents the coronal virtual plane used for alignment, whereas the yellow line represents the axial virtual plane (shown for reference but not used during the alignment process). During alignment, the coronal virtual plane is oriented to match the knife trace and positioned to pass through the corresponding column pair. (B) During alignment, each MRI coronal slice is visually matched to the corresponding blockface photograph before proceeding to the next slice. (C) As alignment progresses, previously matched knife traces are marked with thin blue dashed lines, whereas the current slice position is indicated by a thicker blue dashed line. The pink coronal plane shows the current MRI reconstruction plane, aligned with the cutting orientation. The yellow line indicating the axial plane is shown for reference but not used during the alignment process. (D) The visual match between MRI and blockface images is confirmed for each slab before advancing to the next cutting plane.

Our method aligned ex vivo MRI with blockface photographs of brain slabs using a fiducial‐based approach. In typical fiducial registration—commonly used in neurosurgery—distinct anatomical or artificial markers served as fixed reference points across imaging modalities to ensure precise alignment [23]. In our protocol, the cylindrical columns of the coronal cutting guides served this role. These columns were uniquely positioned and easily distinguishable—appearing white in blockface photographs and dark in T2‐SPACE MR images—allowing them to function as reliable fiducial markers (Figure 5). We assumed that the knife, when guided between column pairs during slicing, followed a coronal virtual plane (pink lines in Figure 5A,C) normal to the embedding medium. Accordingly, the same plane could be reconstructed virtually in the acquired MR images by identifying the corresponding knife trace between the same column pair.

**FIGURE 6:**
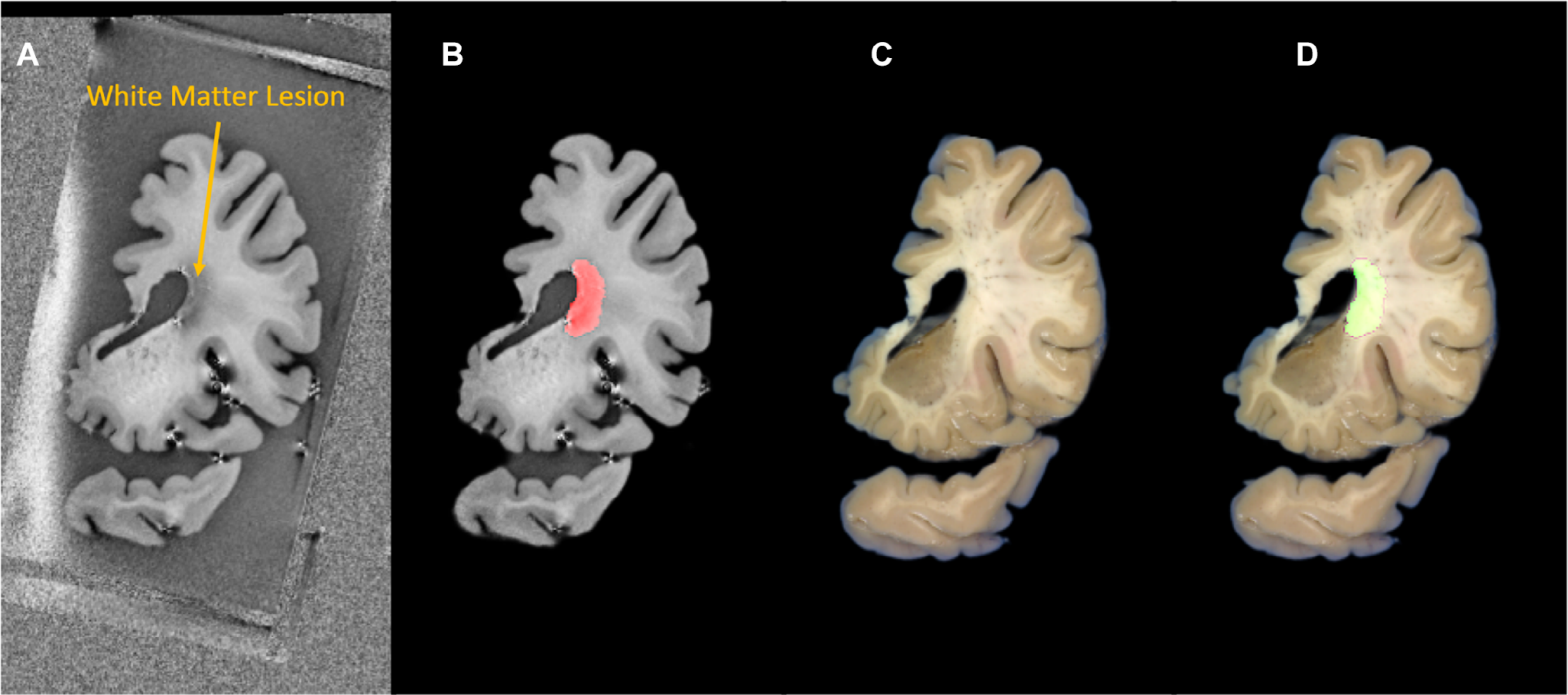
Detection of white matter lesion (arrow) on T1‐weighted MRI with corresponding blockface photograph. (A) T1‐weighted MR image serving as input for our in‐house deep learning model. (B) Automated lesion segmentation mask overlaid on the MR image. (C) Corresponding blockface photograph after image registration and alignment. (D) Lesion mask transferred from MRI to the aligned blockface photograph for histological sampling guidance. The white matter lesion was automatically segmented from the T1‐weighted MRI using our deep learning model, and the resulting mask was subsequently registered to the blockface photograph to guide precise histological sampling.

To perform this alignment, we used the RadiAnt Digital Imaging and Communications in Medicine Viewer (v2022.1.1, 64‐bit) with 3D MPR capabilities. The T1‐MP2RAGE (Figure 6) and T2‐SPACE MR images were loaded into the viewer, and the MPR tool was used to interactively adjust the slicing plane. A corresponding blockface photograph—specifically one showing the first knife trace—was loaded and visually compared. The virtual cutting plane in the MRI was then adjusted to match the knife trace in the photograph, ensuring that the reconstructed MRI slice passed through the same column landmarks. This process was repeated for subsequent coronal tissue slab cuts. Following alignment, white matter lesions were automatically segmented on the MRI (Figure 6A,B), and the resulting labels were overlaid on the corresponding blockface images (Figure 6C,D) to guide tissue sampling.

## 3 Results

We showcase the alignment results for two postmortem human brains following the workflow illustrated in Figure 1  The brains were first embedded in agar within the imaging container. Efforts were made to minimize air bubbles in the agar prior to solidification. Despite differences in brain size, the embedding medium held each brain securely in place, and no visible motion artifacts were observed during scanning. Notably, the workflow and pulse sequences remained consistent across specimens.

**FIGURE 7:**
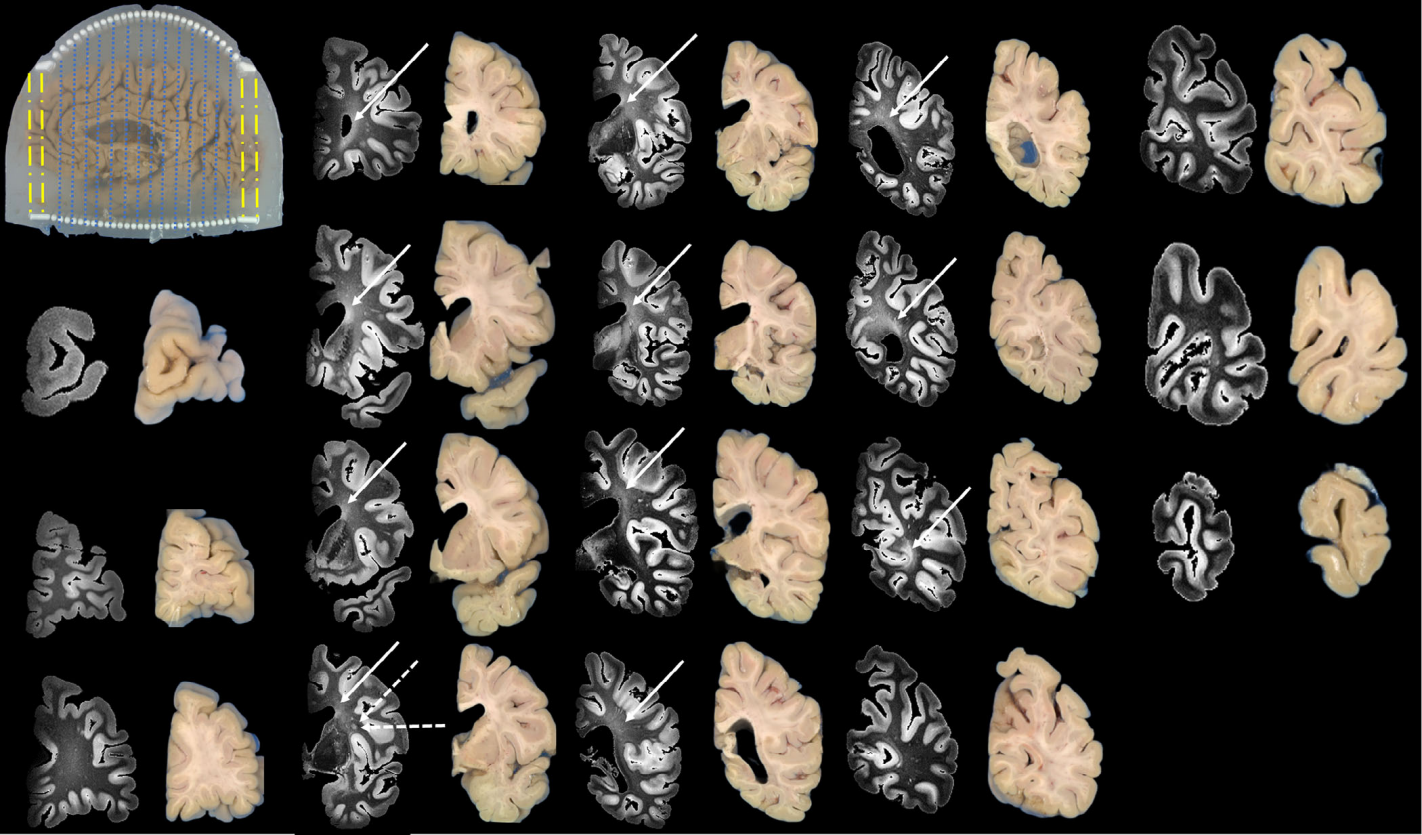
Alignment of T2‐SPACE with blockface photos of the brain sample 1. The yellow dashed lines indicate that the first two and last two slices are cut freehand without the cutting guides, and the blue dotted lines indicate that the slices were cut with the cutting guide. The white solid arrows point to the locations of periventricular lesions, and the white dashe arrows point to the location of the deep white matter lesion.

**FIGURE 8:**
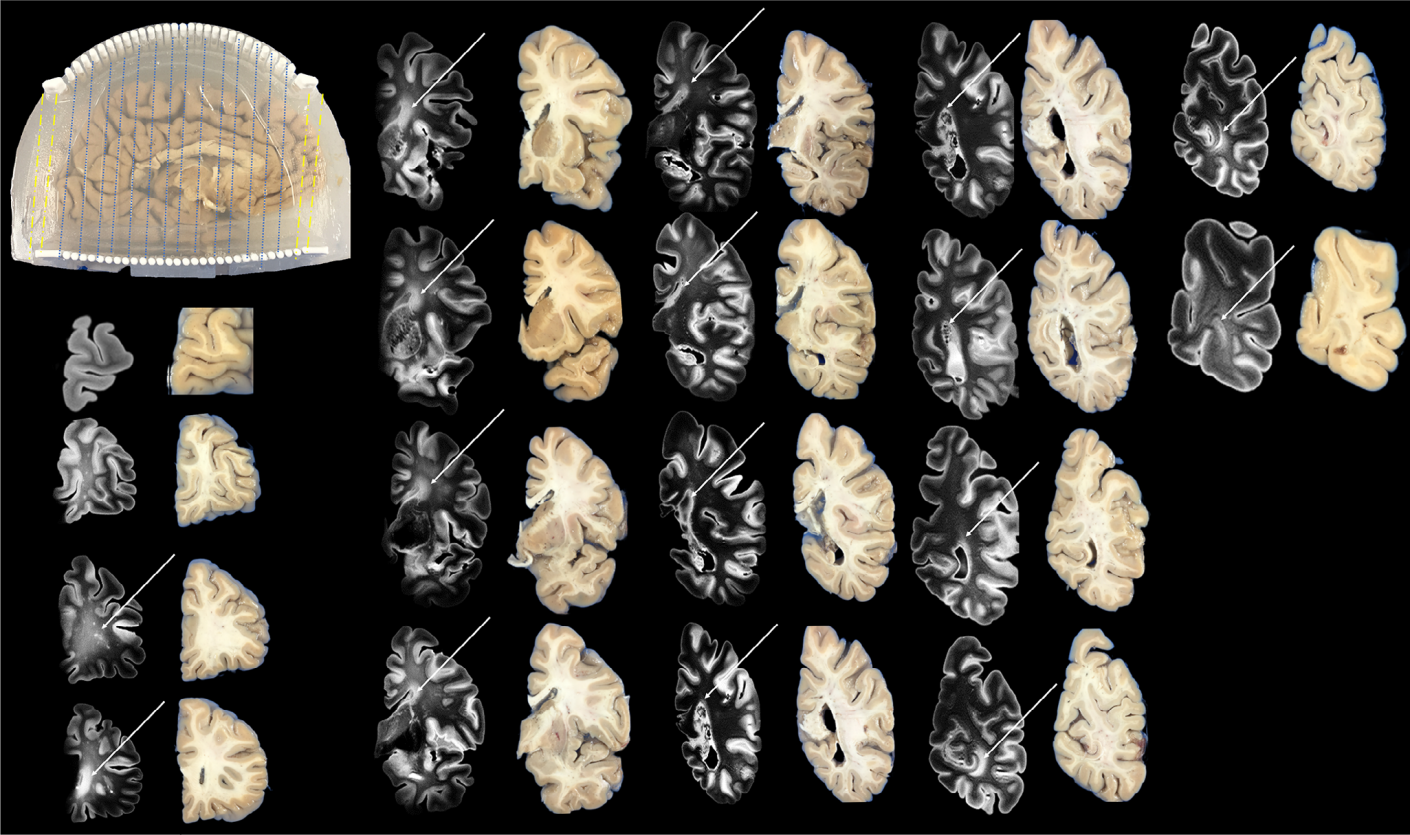
Alignment of T2‐SPACE with blockface photos of the brain sample 2. The dashed yellow lines indicate that the first two and last two slices are cut freehand without the cutting guides, and the blue dotted lines indicate that the slices are cut with the cutting guide. The white solid arrows point to the locations of periventricular lesions.

Brain 1 (Figure 7) was sectioned into 18 coronal slabs, 14 using the coronal cutting guides (dashed blue lines) and 4 freehand (dashed yellow lines). The freehand slabs corresponded to brain regions extending beyond the cutting guide coverage, necessitating manual cuts while maintaining parallel orientation to the guide‐assisted slabs. Similarly, Brain 2 (Figure 8) was divided into 18 coronal slabs, with 14 using coronal cutting guides (dashed blue lines) and 4 freehand (dashed yellow lines). Following sectioning, T2‐weighted SPACE images were aligned to their corresponding blockface photographs. Alignment of all 14 guide‐assisted slabs typically required ∼30 min per brain, using the University of Pittsburgh Center for Research Computing and Data resources. In contrast, the four non‐guided coronal slabs required substantially more time (about an hour each) due to increased variability. For both brains, anterior slab surfaces were successfully matched with their corresponding MRI coronal slices across the entire left hemisphere. Successful matching was determined by visually confirming alignment between fiducial landmarks (cutting guide columns) and corresponding anatomical features (e.g., cortical structures and ventricular boundaries) in both the MRI slices and blockface photographs. White matter lesions were observed in both brains, spanning the periventricular regions from anterior to posterior (solid white arrows, Figures 7 and 8). Additionally, Brain 1 exhibited two distinct deep white matter lesion foci (dashed white arrows, Figure 7).

## 4 Discussion

In this work, we introduced a comprehensive, cost‐effective workflow designed for precise MRI‐guided histological sampling of white matter lesions. By integrating agar sucrose embedding, reusable 3D‐printed cutting guides, ultrahigh‐field 7 T MRI acquisition, and semiautomated alignment methods, our workflow mitigated inhomogeneity imaging artifacts and achieved precise MRI‐histology alignment accuracy.

The agar‐sucrose embedding medium provided exceptional adaptability for various brain sizes by controlling the volume of embedding required based on individual brain volumes. Smaller brains utilized more agar volume, whereas larger brains required less. Once solidified, the agar firmly attached itself to the brain, cutting guides, and container walls, creating a single, immovable body during MRI scanning, significantly minimizing motion artifacts. This approach enabled repeated use of the same container and cutting guides across multiple brains without requiring individualized guides, substantially reducing the cost, especially critical for large‐scale studies.

Reusable, cost‐effective cutting guides played an integral role in the reproducibility of our workflow. Initially, components were printed using high‐density acrylonitrile butadiene styrene filament; however, issues with flexing and splitting between layer lines of the columns during sectioning led us to transition to polycarbonate, which improved layer adhesion and increased stiffness, tensile strength, and impact resistance. During brain sectioning, the agar embedding secured the cutting guides and brain firmly together, preventing sliding and allowing accurate, stable slicing.

The total material and 3D printing cost for one complete set of enclosure, cutting guide, and sealing lid is approximately $60.00 when using polycarbonate filament. With each set lasting approximately 30 brains, the reusable design reduces the effective per‐specimen cost to just $2.00 per brain. Our workflow's alignment precision benefits significantly from using cutting guide columns as landmarks for matching MPR planes to the physical knife traces. This efficient alignment process, typically achievable in less than an hour, streamlines the workflow significantly. Although matching was based on qualitative visual assessment, future work could incorporate quantitative metrics to further validate alignment accuracy. Furthermore, our container is designed to fit precisely within the inner dimensions of the head coil, maximizing the space available for postmortem brain loading. Remarkably, none of the brains that we have imaged require exclusion, highlighting the container's effectiveness in accommodating various brain sizes.

Although our agar‐sucrose embedding provides excellent structural support, it occasionally introduces air bubbles, leading to susceptibility artifacts, primarily affecting GRE‐based imaging near the sulci and hippocampal regions. Despite technique optimization, these bubbles remain challenging to eliminate fully. Additionally, the reliance on agar solidification necessitates careful pre‐solidification orientation planning, resulting in slight variability in the orientation of the cutting plane across specimens. The cutting guides do not span the entire brain, requiring manual freehand cutting in some areas, which increases alignment complexity and duration. Although container placement within the head coil is highly consistent, variability in brain positioning could slightly affect B1+ magnetic field distribution.

In conclusion, we develop and validate a systematic, MRI‐guided workflow for the precise identification and sampling of white matter lesions in postmortem brains using UHF MR imaging and 3D‐printed cutting guides. Our method consists of brain embedding, high‐resolution MRI acquisition, guided sectioning, and fiducial‐based alignment. The cutting guides significantly improve sectioning consistency. The optimized agar‐sucrose embedding medium minimizes dielectric mismatches and motion artifacts, enhancing imaging quality.

## Conflicts of Interest

The authors declare no conflicts of interest.
